# Temperature Field, Flow Field, and Temporal Fluctuations Thereof in Ammonothermal Growth of Bulk GaN—Transition from Dissolution Stage to Growth Stage Conditions

**DOI:** 10.3390/ma16052016

**Published:** 2023-02-28

**Authors:** Saskia Schimmel, Daisuke Tomida, Tohru Ishiguro, Yoshio Honda, Shigefusa F. Chichibu, Hiroshi Amano

**Affiliations:** 1 Crystal Growth Lab, Materials for Electronics and Energy Technology (i-MEET), Friedrich-Alexander-Universität Erlangen-Nürnberg, 91058 Erlangen, Germany; 2 Institute of Materials and Systems for Sustainability, Nagoya University, Nagoya 464-8601, Japan; 3 Electron Devices (LEB), Friedrich-Alexander-Universität Erlangen-Nürnberg, 91058 Erlangen, Germany; 4 Institute of Multidisciplinary Research for Advanced Materials, Tohoku University, Sendai 980-8577, Japan

**Keywords:** ammonothermal, gallium nitride, crystal growth, numerical simulation, computational fluid dynamics, natural convection, buoyancy, conjugated heat transfer, solvothermal, hydrothermal

## Abstract

With the ammonothermal method, one of the most promising technologies for scalable, cost-effective production of bulk single crystals of the wide bandgap semiconductor GaN is investigated. Specifically, etch-back and growth conditions, as well as the transition from the former to the latter, are studied using a 2D axis symmetrical numerical model. In addition, experimental crystal growth results are analyzed in terms of etch-back and crystal growth rates as a function of vertical seed position. The numerical results of internal process conditions are discussed. Variations along the vertical axis of the autoclave are analyzed using both numerical and experimental data. During the transition from quasi-stable conditions of the dissolution stage (etch-back process) to quasi-stable conditions of the growth stage, significant temperature differences of 20 K to 70 K (depending on vertical position) occur temporarily between the crystals and the surrounding fluid. These lead to maximum rates of seed temperature change of 2.5 K/min to 1.2 K/min depending on vertical position. Based on temperature differences between seeds, fluid, and autoclave wall upon the end of the set temperature inversion process, deposition of GaN is expected to be favored on the bottom seed. The temporarily observed differences between the mean temperature of each crystal and its fluid surrounding diminish about 2 h after reaching constant set temperatures imposed at the outer autoclave wall, whereas approximately quasi-stable conditions are reached about 3 h after reaching constant set temperatures. Short-term fluctuations in temperature are mostly due to fluctuations in velocity magnitude, usually with only minor variations in the flow direction.

## 1. Introduction

The low-pressure acidic ammonothermal (LPAAT) method is considered one of the most promising technologies for the scalable production of bulk GaN substrates with high structural quality and at low cost [1,2,3]. Therefore, it represents a promising route for overcoming the lack of native substrates, which currently limits the development of GaN-based electronic devices [4]. Although blue and white light-emitting diodes realized on foreign substrates have already revolutionized lighting technology [5,6], further applications among others in power electronics [7] demand better structural quality and benefit more from the use of native substrates. The lower defect densities achievable with native substrates enable the realization of lower off-state leakage currents, which in turn improves the energy efficiency in energy conversion applications [8]. However, for widespread application to tap the full potential of GaN-on-GaN technology, wafer prices would need to decrease significantly [8] in relation to the cost of the saved energy, and the availability needs to improve. For both, efficient scale-up of bulk GaN growth technologies such as the ammonothermal method is essential. To provide an avenue for efficient scale-up and high yield in production, the development of numerical models of the growth process is expected to be very important, especially considering the great technical difficulty of experimental access to ammonothermal autoclaves. Purely data-based empirical models or hybrid models may eventually become effective for process improvement and control once the method is used industrially at a large scale and enough experimental data are generated. At the present stage, however, physics-based numerical simulations [9] and in situ monitoring technologies [10,11,12] are more feasible and are also particularly suitable for advancing fundamental understanding. Besides the application to GaN crystal growth, an improved fundamental understanding of ammonothermal crystal growth conditions would also facilitate the targeted application of the ammonothermal method as a tool for explorative synthesis [13] of various nitride materials and the development of single crystal growth processes for these materials. In other crystal growth processes, data generated via physics-based numerical simulations have also proven highly valuable as input data for machine learning models. In SiC solution growth, for instance, the tremendously accelerated prediction of growth conditions has been demonstrated [14]. In the directional solidification of silicon, the accelerated optimization of growth conditions has been presented [15]. Therefore, once the physics have been clarified and sufficiently accurate models have been developed on that basis, machine learning from numerical simulation data may provide a pathway to realize computationally efficient compact models for ammonothermal crystal growth.

In this study, we investigate the quasi-stationary conditions in ammonothermal growth of GaN in the geometrical configuration used for growth under retrograde solubility conditions, which applies to the use of NH_4_F mineralizer [1] as well as ammonobasic growth [16]. Contrary to most prior studies, we do not use heater-long fixed temperatures as a thermal boundary condition. Instead, a temperature distribution based on a simulation, including the growth furnace, is used, which is closer to the experimental conditions according to recent studies [17,18].

A detailed analysis of flow stability and temperature fluctuations at different probing locations is presented. A lack of spatial and temporal uniformity of material transport to the growth front is suspected of causing the formation of defects, and therefore the spatial and temporal uniformity of fluid flow is also thought to affect defect formation [19].

To shine a first light on the conditions during the initial stages of the crystal growth process, the effect of inverted set temperatures is investigated approximately. Such an inverted temperature gradient is applied during temperature ramp-up by experimentalists and is intended for back-etching of the seed crystals [20], hereby likely removing impurities as well as sub-surface damage from cutting and grinding (if present) and modifying the surface morphology due to defect-selective etching, which occurs in both ammonobasic [21] and ammonoacidic [22] solutions. Besides quasi-stable temperature and fluid flow conditions during etch-back and growth, we also analyze the conditions occurring temporarily while changing set temperatures from etch-back to growth conditions. In addition, we analyze how quasi-stable conditions are re-established after the set temperatures of the growth stage have been reached and are kept constant.

## 2. Materials and Methods

### 2.1. Numerical Simulation

In our previous study [17], we examined the temperature distribution along the outer wall of an ammonothermal autoclave inside a resistively heated furnace with two separately controlled heaters (the thermocouples for heater power control being located at the outer autoclave wall, one for each heater). Regarding the geometry, materials properties, and the use of the LVEL (Length-VELocity) turbulence model, the models in this study were set up in full analogy to the type C cases in the mentioned previous study. Note that although the occurrence of turbulent flow was not prevented by the selection of the flow model, laminar flow can likewise occur, depending on the location and the rate of thermal boundary condition change. In preliminary numerical experiments with this model, we observed that the flow cell structure between the baffle and uppermost seed does change if the flow is forced to be laminar (also see our previous publications [9] and [17] for further discussion of the question of laminar versus turbulent flow in ammonothermal crystal growth). In addition, we intentionally kept the model very similar to the one in the previous study because otherwise, it would not be reasonable to use the results of the previous study for boundary condition definition. Minor modifications with regard to the type C cases in our previous study [17] and model variants are detailed below. Note that two versions with different seed thicknesses were studied. While we applied a seed thickness of 3 mm (thereof 1.5 mm inside the simulation domain) in our previous simulation [17], a seed thickness of 10 mm (thereof 5 mm inside the simulation domain) was used in the present study unless otherwise stated. The reason for this is that we preferred to first obtain results for the larger seed thickness because these can easily be used as initial conditions for further studies with thinner seeds. This does not work as easily if performed in the opposite order (likely due to issues with non-zero velocity values inside solids if enlarging the seed size rather than shrinking it).

In Table 1, the geometry is described by the positions of the centers of the components and their dimensions.

**Figure 1:**
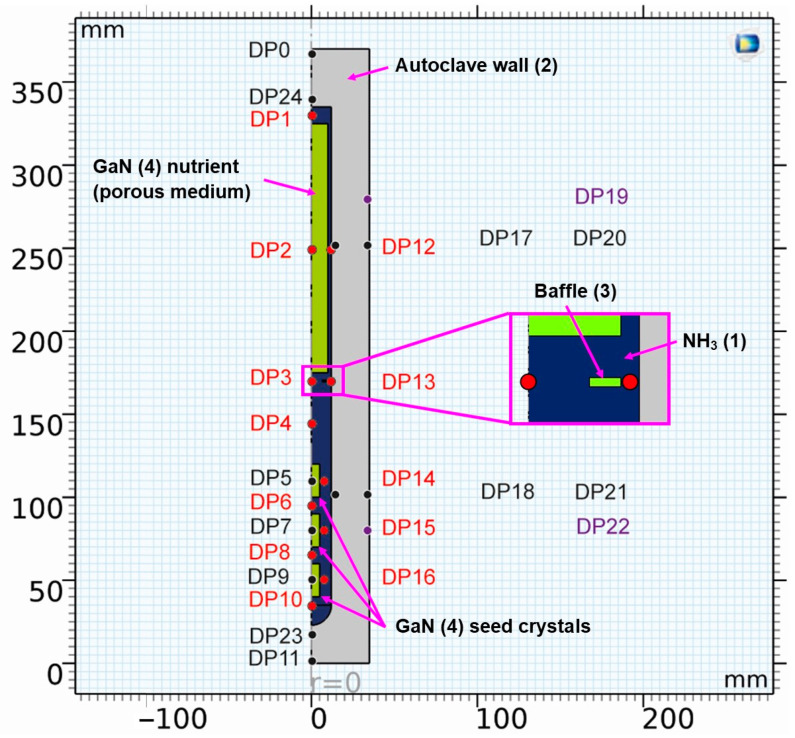
Geometry of the model with location of domain point probes (see Table 2 for coordinates thereof and for a description of the chosen locations). The color coding of the labels is as follows: probes in the fluid (red), probes in solids (black and purple), and probes at the location of control thermocouples in an experimental setup (purple). The components are also labeled, including a number that refers to the material (see Table 3, Table 4 and Table 5 for further information on materials).

The geometry, alongside information on probing locations for the evaluation of temporal fluctuations, is depicted in Figure 1.

To complement the information in Figure 1, the domain probe locations are listed and described in Table 2.

A list of the components labeled with numbers in Figure 1, together with a description of the materials they consist of, can be found in Table 3. Note that, in the case of GaN, bulk GaN properties were assumed for the seeds, whereas the GaN nutrient was represented as a porous medium with a porosity of 0.7 and a permeability of 0.6352 mm^2^.

Note that ammonia is in its supercritical state during all studied stages of a growth run. The amount of ammonia inside the autoclave does not undergo any externally controlled changes during a growth run. While chemical reactions, as well as leakages, can cause changes in ammonia density during a growth run in experimental work, neither of these possible effects was implemented in the numerical model. For these reasons, the same mean density of the fluid was used regardless of the temperatures applied (that is, neglecting possible effects of solutes on the thermophysical properties of the fluid, thus also neglecting solutal convection). The temperature-induced variations of ammonia density were, therefore, the only driving force for convection and implemented using Boussinesq approximation (Equation (1)), with a reference temperature $T_{ref}$ = 293.15 K and a reference density $\rho_{ref}$ = 233.950 kg/m³. Therein, $\alpha_{v}$ is the volumetric thermal expansion coefficient.

(1) $$\rho -\rho_{ref}=-\rho_{ref}\alpha_{v}\left(T-T_{ref}\right)$$

The density of 233.950 kg/m³ corresponds to the density of supercritical ammonia at 100 MPa at a temperature of 554.0 °C (mean value of fluid temperatures inside the autoclave at growth stage conditions). These conditions mimic those typically used in the experimental LPAAT method. Temperature-dependent data of supercritical ammonia taken from the NIST database [23] were used. Beyond 426.9 °C, data for thermal conductivity, heat capacity at constant pressure, density, and the ratio of specific heats were extrapolated linearly, whereas a constant extrapolation was used for the dynamic viscosity. The materials properties of supercritical ammonia at 426.9 °C are listed in Table 4.

**Table 4:** Fluid properties of supercritical ammonia at 426.9 °C as obtained from NIST database [23].

Material	ρ/kg/m^3^	ν/m^2^/s	c_p_/J/(kg∙K)	k/W/(m∙K)	α/1/K
NH_3_ (1)	233.950	1.570 × 10^−7^	4216.5	0.16224	2.631 × 10^−3^

**Table 5:** Materials properties of the solid components. For the autoclave walls, the in-build materials property definitions for the nickel base superalloy Haynes R-41 of the Comsol Multiphysics materials library were used.

Material	ρ/kg/m^3^	c_p_/J/(kg∙K)	k/W/(m∙K)
Autoclave wall (2)	8301.112+0.1012931*T^1-0.001639246*T^2+8.064631E-7*T^3+1.085603E-8*T^4-1.915217E-11*T^5 (from 33 to 366 K) 8335.344-0.3077835*T^1+6.941174E-5*T^2-9.845641E-8*T^3 (from 366.0 to 1366.0 K)	474.575639-0.12868256*T^1+1.2284019E-4*T^2+2.01913857E-7*T^3-4.98757486E-11*T^4 (from 293.0 to 1483.0 K)	3.843196+0.01841176*T^1 (from 293.0 to 1173.0 K)
Baffle (3)	10200.00	250.00	138.00
Bulk GaN (4)	6150.00	518.41	100.13

For all solid components, the properties of the materials are listed in Table 5.

The remaining governing equations are given in the following, with the subscript *w* indicating quantities at interfaces to solids (walls).

The conservation of mass is expressed by the continuity equation (Equation(2)):

(2) $$\rho \nabla \cdot \vec{u}=0$$

Equation (3) describes the conservation of momentum for the flow of an incompressible fluid. The term $\left(\rho -\rho_{ref}\right)\vec{g}$ therein represents the buoyancy force per unit volume, and the pressure gradient is equal to $\nabla \cdot \left(-p\vec{I}\right)$. The vector $\vec{u}$ contains the velocity components *u* and *w*, t is the time, p the pressure, $\vec{I}$ the identity matrix, μ the dynamic viscosity of the fluid, $\mu_{T}$ the turbulent viscosity.

(3) $$\rho \frac{\partial \vec{u}}{\partial t}+\rho \left(\vec{u}\cdot \nabla \right)\vec{u}=\nabla \cdot \left[-p\vec{I}+\vec{K}\right]+\left(\rho -\rho_{ref}\right)\vec{g}\;\mathrm{with}\;\vec{K}=\left(\mu +\mu_{T}\right)\left(\nabla \vec{u}+{\left(\nabla \vec{u}\right)}^{T}\right)\;\mathrm{with}\;\mu_{T}=\mu \left(\frac{dl_{w}^{+}}{du^{+}}-1\right)$$

The dimensionless distance from the wall in the LVEL model $l_{w}^{+}$ is defined by Spalding’s law of the wall [24] (Equation (4)):

(4) $$l_{w}^{+}=u^{+}+\frac{1}{E}\left(e^{\kappa u^{+}}-1-\kappa u^{+}-\frac{{\left(\kappa u^{+}\right)}^{2}}{2}-\frac{{\left(\kappa u^{+}\right)}^{3}}{6}-\frac{{\left(\kappa u^{+}\right)}^{4}}{24}\right)$$

Equation (5) describes the local Reynolds number at the interfaces to solids, with the dimensionless quantity $u^{+}$ being the local flow speed divided by the friction velocity and $l_{w}^{+}$ being the dimensionless distance from the wall.

(5) $$Re_{w}=\frac{\rho \left|\vec{u}\right|l_{w}}{\mu }=\frac{\left|\vec{u}\right|}{u_{\tau }}\cdot \frac{\rho u_{\tau }l_{w}}{\mu }=u^{+}l_{w}^{+}$$

The wall distance $l_{w}$ is given by Equation (6):

(6) $$l_{w}=\frac{1}{G}-\frac{l_{ref}}{2}$$

A modified version of the Eikonal equation (in which the dependent variable is $G=1/l_{w}$ instead of the exact distance to the closest wall $l_{w}$) is used to describe the reciprocal wall distance G (Equation (7)), with the smoothing parameter $\sigma_{w}$ being equal to 0.2:

(7) $$\nabla G\cdot \nabla G+\sigma_{w}G\left(\nabla \cdot \nabla G\right)=\left(1+2\sigma_{w}\right)G^{4}$$

The dimensionless effective viscosity $\nu^{+}$ can be obtained from Equation (8):

(8) $$\nu^{+}=1+\frac{\kappa }{E}\left(e^{\kappa u^{+}}-1-\kappa u^{+}-\frac{{\left(\kappa u^{+}\right)}^{2}}{2}-\frac{{\left(\kappa u^{+}\right)}^{3}}{6}\right)$$

No slip wall conditions are used (Equation (9)):

(9) $${\vec{u}|}_{l_{w}=0}=\vec{0}$$

The flow in the porous medium is described as follows by the Brinkman equations (Equations (10) and (11)), with $Q_{m}$ being the mass source term:

(10) $$\rho \nabla \cdot \vec{u}=Q_{m}=\vec{0}$$

With the porosity $\epsilon_{p}$ and the local permeability κ:

(11) $$\frac{1}{\epsilon_{p}}\rho \frac{\partial \vec{u}}{\partial t}+\frac{1}{\epsilon_{p}}\rho \left(\vec{u}\cdot \nabla \right)\vec{u}\frac{1}{\epsilon_{p}}=\nabla \cdot \left[-p\vec{I}+\vec{K}\right]-\left(\mu \kappa^{-1}+\beta \rho \left|\vec{u}\right|+\frac{Q_{m}}{\epsilon_{p}^{2}}\right)\vec{u}+\left(\rho -\rho_{ref}\right)\vec{g}$$

With $Κ=\mu \frac{1}{\epsilon_{p}}\left(\nabla \vec{u}+{\left(\nabla \vec{u}\right)}^{T}\right)-\frac{2}{3}\mu \frac{1}{\epsilon_{p}}\left(\nabla \cdot \vec{u}\right)\vec{I}$

The Forchheimer coefficient β is calculated from the Forchheimer parameter $c_{F}$ and the local permeability κ (Equation (12)):

(12) $$\;\beta =\frac{c_{F}}{\sqrt{\kappa }}$$

The energy equation in the temperature form (Equation (13)) is as follows. Therein, Q represents the heat supplied to the system and the vector $\vec{q}$ describes the conductive heat flux, $C_{p}$ is the specific heat capacity and k is the thermal conductivity.

(13) $$\rho C_{p}\frac{\partial T}{\partial t}+\rho C_{p}\vec{u}\cdot \nabla T+\nabla \cdot \vec{q}=Q\;$$

The conductive heat flux vector therein is defined by Equation (14), Fourier’s law of conduction:

(14) $$\vec{q}=-k\nabla T=0$$

Two model versions with thermal boundary conditions constant over time were used to investigate the quasi-stable conditions during the etch-back and growth stages of a crystal growth experiment. Model 550_650 uses the temperature distributions at the outer autoclave wall as obtained in model A3 in the previous study [17] (model A3 included the furnace as well as internal solids). It represents an ammonothermal setup with an uninsulated autoclave head, with the top heater set to 550 °C and the bottom heater set to 650 °C.

**Figure 2:**
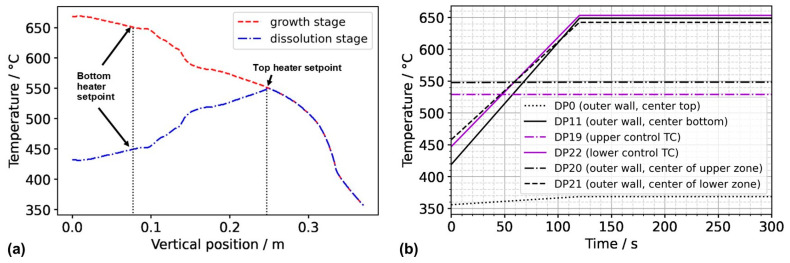
(**a**) Thermal boundary conditions imposed along the vertical outer wall of the autoclave for Model 550_650 (growth stage) and Model 550_450 (dissolution stage of the growth experiment). (**b**) Temperatures at domain probes located at the outer wall over time. The abbreviation TC stands for thermocouple, referring to the position thereof in the experimental setup.

To obtain a first insight into the conditions in the case of inverted thermal gradient in a computationally efficient way, the following assumptions were used to obtain approximate but reasonably realistic thermal boundary conditions for this case, which we term Model 550_450. We assume that since the set temperature of the top heater, as well as the uninsulated condition of the autoclave head, is identical to model A3 in our previous study [17], autoclave wall temperatures of the upper part of the autoclave down to the position of the control thermocouple of the top heater can be approximated as the outer wall temperature distribution extracted from the results of model A3. This is likely not perfectly accurate, as a measurable influence of convective heat transfer on the wall temperatures of the autoclave head has been observed in an experimental study of the first author [10]. However, this influence is likely less pronounced in the configuration studied here, as the presence of the porous medium is expected to lead to lower flow velocities. For the vertical temperature distribution from the location of the control thermocouple of the top heater to the bottom of the setup, we assume that a near-linear temperature profile will develop in analogy to the one obtained in case A3 in our previous study [17], albeit with different absolute temperatures to account for the different set temperature of the bottom heater (450 °C). The temperature distributions imposed along the vertical autoclave wall are plotted in Figure 2 for Model 550_650 as well as Model 550_450. The temperature distribution along the bottom autoclave wall is assumed to show the same characteristics as in A3 but was shifted so that the vertical and horizontal autoclave wall temperature distributions share the same temperature at the bottom outer edge of the autoclave wall.

The boundary conditions were based on the temperature profiles extracted from the previous study and defined as a function of coordinates, the time *t,* and the duration of the set temperature inversion process Δt as follows. For the upper outer autoclave wall (z = 370 mm), the thermal boundary conditions were defined by the function in Equation (15):

(15) $$T_{W}\left(r,t,z=370\;\mathrm{mm}\right)=T_{w}\_GS\left(r,z=370\;\mathrm{mm}\right)-\frac{T_{w}\_GS\left(r,\;z=370\;\mathrm{mm}\right)-T_{w}\_DS\left(r,\;z=370\;\mathrm{mm}\right)}{\Delta t}\left|t-\Delta t\right|$$

In the same way, the function describing the thermal boundary condition at the bottom outer autoclave wall was defined by Equation (16):

(16) $$T_{W}\left(r,t,\;z=0\;\mathrm{mm}\right)=T_{w}\_GS\left(r,\;z=0\;\mathrm{mm}\right)-\frac{T_{w}\_GS\left(r,\;z=0\;\mathrm{mm}\right)-T_{w}\_DS\left(r,\;z=0\;\mathrm{mm}\right)}{\Delta t}\left|t-\Delta t\right|$$

The function describing the time-dependent thermal boundary conditions for the vertical outer wall is defined in the same way (Equation (17)):

(17) $$T_{W}\left(z,t,\;r=35\;\mathrm{mm}\right)=T_{w}\_GS\left(z,\;r=35\;\mathrm{mm}\right)-\frac{T_{w}\_GS\left(z,\;r=35\;\mathrm{mm}\right)-T_{w}\_DS\left(z,\;r=35\;\mathrm{mm}\right)}{\Delta t}\left|t-\Delta t\right|$$

**Figure 3:**
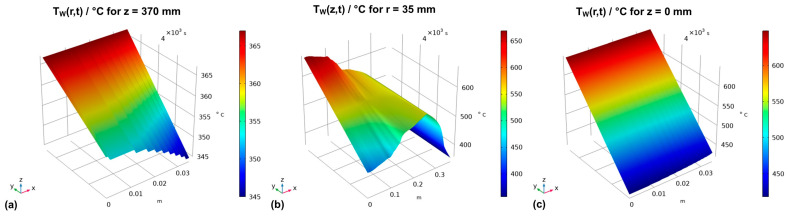
Thermal boundary conditions over time for (**a**) the horizontal upper outer autoclave wall, (**b**) the vertical outer autoclave wall, and (**c**) the horizontal bottom outer autoclave wall.

The resulting thermal boundary conditions as a function of the coordinates and time are visualized by the graphs in Figure 3, where subfigures (a), (b), and (c) represent the thermal boundary conditions at the upper outside wall, the vertical outside wall, and the lower outside wall, respectively.

**Figure 4:**
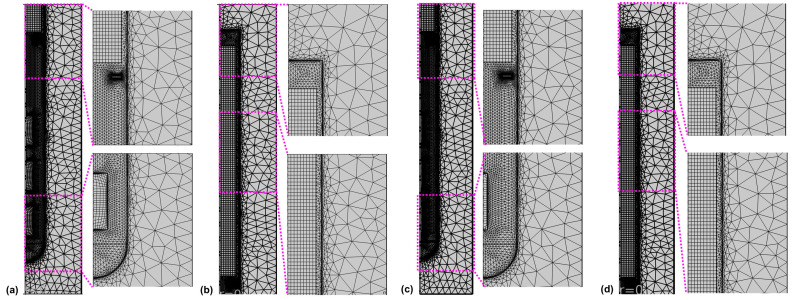
Mesh in (**a**,**c**) the lower and (**b**,**d**) the upper part of the autoclave for the model versions with thick seeds (**a**,**b**) and thin seeds (**c**,**d**).

Calculations were done using the commercial software Comsol Multiphysics. A combination of structured and unstructured mesh regions was used, as depicted in Figure 4.

The parameters used to create the mesh are given in Table 6. Four boundary layers with a stretching factor of 1.2 were employed at the interface of the fluid with the autoclave walls, baffle, and seeds, respectively. The thickness of the first boundary layer was set to 0.02 mm. The relative tolerance was set to 0.002 for Model 550_650, whereas a relative tolerance of 0.050 was applied for the inverted temperature Model 550_450 to facilitate convergence. The convergence difficulties observed when initially using the same relative tolerance are thought to be due to the less accurate estimation of thermal boundary conditions in the case of the inverted temperature version of the Model 550_450.

Time steps taken by the solver were constrained to a maximum of 0.005 s to ensure sufficient temporal resolution to resolve fluid flow fluctuations and short-lived small eddies on small time scales. As for the time-stepping method, the implicit backward differentiation formula (BDF) was applied. The model was solved using a fully coupled approach, employing a direct solver (PARallel DIrect Solver, PARDISO for short). Newton’s method was used as the nonlinear method (specifically, Constant (Newton)), with the Jacobian update on every iteration. To avoid memory-related issues as well as the creation of excessively large recovery files, the computation was split into sections of 1000 s of real time. To ensure that any disturbances upon restart would not be part of the evaluated data, the last 900 s of each section were used for evaluation.

To identify whether a quasi-stable state had been reached, the temperatures, as well as the velocity components (where applicable), were plotted over time for all domain point probe locations specified in Figure 1. It should be noted that while it is computationally feasible to analyze most of the temporal changes in temperature and velocity fields using our current model and resources, the following limitations exist and are expected to lead to minor inaccuracies. Firstly, as a physical effect, the rates of temperature and velocity changes (time-averaged, so far as domain probes are located inside the fluid and therefore experience short-term fluctuations) decrease while the system approaches a quasi-stable state. This is expected because the thermal gradients between adjacent system components decrease, especially those between crystals and surrounding fluid. Even after the average temperature of the surrounding fluid has been established inside each seed crystal, slow temperature changes are still observed. This includes sluggish, small changes of fluid temperatures at various locations within the fluid, which also lead to a slow change of temperatures inside the seed crystals. Since the rates of temperature change decrease as quasi-stable conditions are approached, the computational cost in relation to the improvement in accuracy increases. To balance computational cost and accuracy, quasi-stable conditions were determined approximately. The term quasi-stable conditions in this study refer to a state at which the rates of temperature changes have decreased below 0.15 K/min at all domain probe locations. Note that the rates of temperature change at conditions termed quasi-stable were virtually constant at most domain probe locations and below 0.03 K/min at center and gap locations at the vertical position of baffle and center of the nutrient (i.e., DP2, DP12, DP3, DP13). The remaining temperature differences between the centers of the seeds and the domain probe inside the fluid at the same vertical height were 2.2 K, 0.9 K, and 3.0 K for the top, middle, and bottom seeds, respectively. The loss of accuracy due to using an approximately quasi-stable solution is estimated to be in the order of a few Kelvin.

For visualization using animations, arrows with logarithmic scaling were used for both models (arrow placement: Gauss points with the maximum number of 5000, arrow base: tail). For Model 550_450, a scale factor of 300 was used for the arrows visualizing fluid flow. The scale factor was adapted to 100 for Model 550_650 to maintain distinguishable arrows despite the higher flow velocities. In both cases, one frame was exported every 0.02 s, and a frame rate of 50 frames per second was used. Thus, the videos are a real-time representation of the results. The Videos S1–S5 represent a 30 s timespan at quasi-stable dissolution stage conditions for the uppermost region of the autoclave, the middle region of the autoclave (between the baffle and top seed), the region around the top seed, the region around the middle seed, and the region around the bottom seed, respectively. The Videos S6–S10 represent a 30 s timespan at quasi-stable growth stage conditions for the uppermost region of the autoclave, the middle region of the autoclave (between the baffle and top seed), the region around the top seed, the region around the middle seed, and the region around the bottom seed, respectively.

For studying the transition from dissolution to the growth stage, slight modifications were made to improve computational efficiency while maintaining stable convergence (constant time step size taken by the solver). Specifically, the relative tolerance was increased to 0.3, and the maximum time step was increased to 0.01 s. The growth zone set temperature change imposed was 200 K over a time period of 2 h, i.e., 100 K/h.

### 2.2. Experimental Crystal Growth under Similar Conditions

To provide at least some comparison to experimental data, results of an ammonoacidic crystal growth experiment using an NH_4_F mineralizer are reported. The mineralizer concentration was 2.00% (amount of substance: 0.02714 mol) and the crystal growth time (at growth stage set temperature) was 24 h. The initial pressure upon reaching growth stage temperatures was 98.5 MPa and the final pressure before cool-down was 100.3 MPa. The initial amount of polycrystalline nutrients was 35.59 g. GaN seed crystals grown by hydride vapor phase epitaxy (HVPE) were purchased from Mitsubishi Chemical Corporation. Each seed was 10 mm wide and 20 mm long (for thicknesses, see the results section). The vertical positions of seed centers were 15 mm, 35 mm, and 58 mm above the inner bottom wall. The baffle position was 100 mm above the bottom inner wall. The outer diameter of the ring-shaped baffle was 20 mm, and the diameter of the center hole was 4.0 mm. The dimensions of the autoclave were the same as in the numerical model. During etch-back, set temperatures were 500 °C (top heater) and 450 °C (bottom heater), respectively. Etch-back set temperatures were kept constant for 5 h. Within this time, a saturation of the solution can be expected so far as not prevented by transport and redeposition following solubility gradients: In situ monitoring experiments conducted with near-isothermal temperature field showed a saturation of the solution within about 2 h while passing through a temperature range from about 220 °C to 500 °C [21]. Rates of set temperature increase were 100 K/h as in the numerical model. During the growth stage, set temperatures were 575 °C (top heater) and 625 °C (bottom heater), respectively. The thickness of the seed crystal before the experiment was measured with a micrometer. The seed crystal and growth layer thicknesses after the experiments were determined from cross-sectional UV fluorescent micrographs. The post-etch-back mass was estimated based on the remaining seed thickness using the density of GaN (6.15 g/cm³) and the initial seed area (200 mm²).

## 3. Results and Discussion

**Figure 5:**
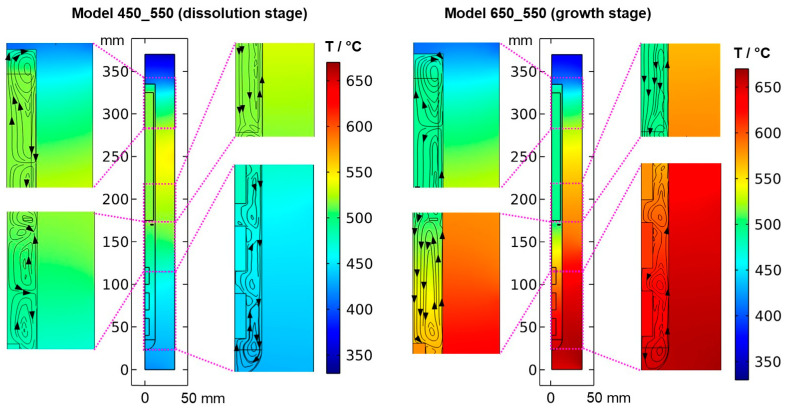
Thermal field in the quasi-stable state during dissolution and growth stage, together with the respective velocity fields visualized by streamlines.

**Figure 6:**
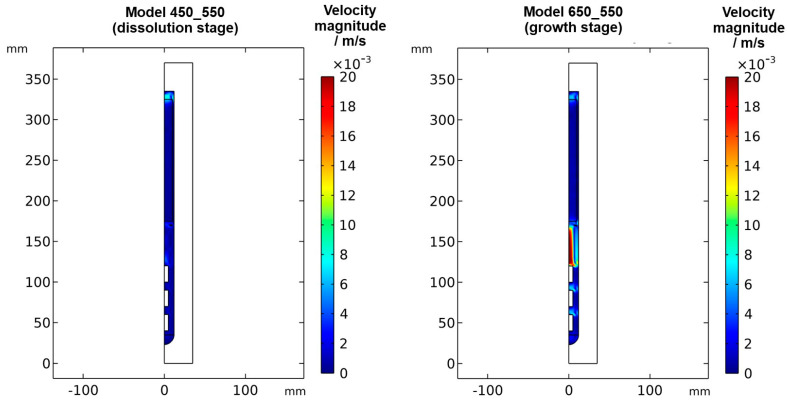
Velocity magnitude at dissolution stage (**left**) and growth stage (**right**) in a quasi-stable state.

**Figure A1:**
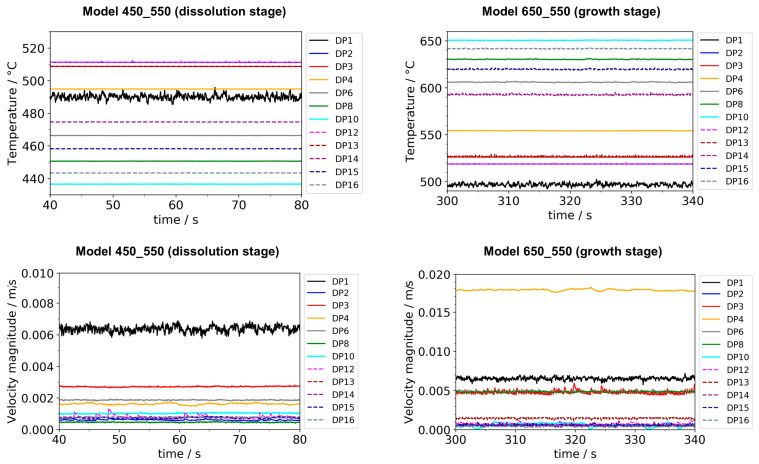
Temperature (**top**) and velocity magnitude (**bottom**) over time for all probe locations in the fluid at dissolution stage (**left**) and growth stage (**right**), respectively. Note the different y-axis scales at growth and dissolution stage. Data were exported with a temporal resolution of 0.05 s. Note that the times shown here should be interpreted only in terms of time range, as they do not relate to the time values elsewhere in this work.

**Figure A2:**
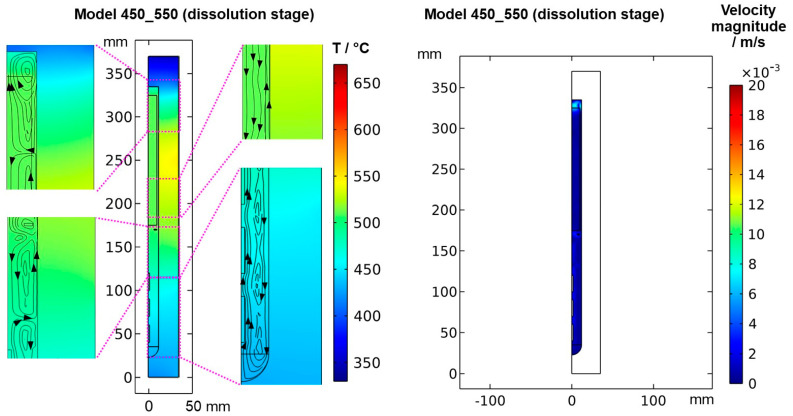
Quasi-stable dissolution stage conditions for the model version with slim seeds: Temperature field with streamlines visualizing fluid flow directions (**left**) and velocity magnitude field (**right**).

To confirm that quasi-stable conditions were reached, plots of temperature and velocity probes at various locations are displayed in Figure A1. For both quasi-stable conditions, the most pronounced fluctuations in flow velocities and temperatures are observed for domain probe DP1 located in-between the top inner wall and the upper end of the nutrient. The general characteristics regarding both temperature and fluid flow fields were confirmed to be similar for both model versions differing in the thickness of seeds (compare the left subfigures of Figure 5 and Figure 6 to Figure A2). This suggests that the internal temperature and fluid flow characteristics are relatively insensitive to moderate changes in crystal dimensions, such as those during etch-back and early crystal growth.

The resulting thermal fields and flow fields for both quasi-stable cases are shown in Figure 5 and Figure 6, respectively. As expected, the inversion of the direction of the thermal gradient along the vertical autoclave wall carries over into the interior, though with noticeable temperature differences between the fluid in the growth zone and the seeds compared to the autoclave wall. Likewise, at the growth stage, a noticeable temperature difference occurs between the nutrient and the autoclave wall for most locations within the nutrient. These effects are thought to be caused by the pronounced natural convection that is present at the growth stage due to the warmer temperatures at the bottom. The conditions observed for both quasi-stable conditions is discussed in Section 3.1. and Section 3.3, respectively. Regardless of the different stages of the process, the temperature is relatively uniform within each seed. This is likely due to the high thermal conductivity of GaN (100.13 W∙m^−1^∙K^−1^) compared to that of supercritical ammonia of the considered density (about 0.16 W∙m^−1^∙K^−1^), which is also a likely additional reason for the rather uniform temperature within the nutrient.

**Figure 7:**
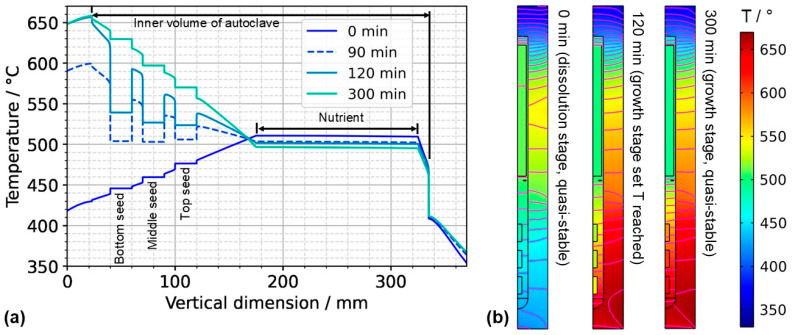
(**a**) Temperature distribution along the centerline for selected points in time. The data labeled with 0 min correspond to quasi-stable conditions at dissolution stage, whereas the data labeled with 120 min correspond to the time at which growth stage set temperatures were reached. The data labeled with 300 min correspond to the time at which quasi-stable conditions at growth stage were reached. The time t = 90 min is also shown, as it represents the time at which all seeds and nutrients have nearly the same temperature. (**b**) Temperature distribution with isotherms for quasi-stable dissolution stage conditions (0 min), at the end of set temperature change (120 min), and for quasi-stable conditions at growth stage (300 min). The temperature difference between isotherms is 10 K, and the range of temperatures is 330 °C to 670 °C.

Expectably, the temperature of each seed is an average of the temperatures within the surrounding fluid (see the times 0 min and 300 min in Figure 7a as well). Consequently, the dissolving and growing GaN surfaces are immersed in a fluid of higher or lower temperature depending not only on the process stage but also on the position within a seed. The question arises of how this inhomogeneity of the thermal gradient between seed and fluid influences crystal growth, especially if seeds with a large dimension in the vertical direction are used. In experimental ammonothermal growth of GaN, the remaining seed after etch-back is regularly found to be thinner at the lower side of the seed, which is in qualitative agreement with this numerical result. Note that there is also a possibility that this effect might be altered if fluid flow in 3D and its time-averaged effects are considered: The degrees of freedom of the fluid flow will have an influence on convective heat transfer within the fluid, and different velocity magnitudes at relevant surfaces such as the autoclave walls will influence the heat transfer coefficient and hence the heat transfer between solids and fluid.

The quasi-stable conditions of etch-back and growth, as well as the conditions occurring temporarily in between, are considered to be important characteristics of the growth process. In Figure 7a, the temperature distribution along the centerline is shown for selected times corresponding to quasi-stable conditions of etch-back, reaching of set temperatures of growth stage, and quasi-stable conditions of growth stage, whereas Figure 7b) shows the respective temperature distributions and isotherms in 2D. Although details are discussed in the respective subsections, this overview already reveals a couple of relevant characteristics.

As for the temperature within the nutrient, it is noteworthy that it *decreases* as the set temperature of the bottom heater is changed from its etch-back value to its (higher) growth stage value, in spite of the constant thermal boundary conditions in the upper part of the model. This can be explained considering the changes in the flow field: By establishing a boundary condition with the bottom of the autoclave being warmer than the nutrient and top region, a strong driving force for natural convection is established, which also leads to a pronounced downward flow through the baffle and towards the top seed (Figure 6). This enhanced convection facilitates heat transfer from the cold top to the nutrient, which apparently dominates over the increased heat transfer from the warm bottom to the nutrient zone.

Within the growth zone, rather high thermal gradients occur at the seed–fluid and the fluid–wall interface under certain conditions. These are most pronounced under transient conditions during fast changes in thermal boundary conditions (t = 120 min in Figure 7b). Such high thermal gradients occur only at interfaces and only while a significant driving force for natural convection is present (t = 120 min and 300 min in Figure 7b). They are therefore thought to be closely related to natural convection. In the case of the fluid–wall interface, the inner wall of the autoclave is heated by heat conduction in the autoclave wall and thereby keeps a temperature similar to the temperature imposed at the outer wall. The natural convection, however, transports rather cool fluid from the nutrient region and top of the autoclave into the growth zone, which temporarily overcompensates the heating of the fluid by the warm inner wall. The high thermal gradient at the fluid-wall interface is, therefore, explainable.

Even higher thermal gradients are temporarily observed at the seed–fluid interfaces. Major temperature differences occur around the time at which set temperatures of growth stage have been reached, but the respective quasi-stable conditions have not been established yet. In addition to the effect of natural convection described for the fluid–wall interface in the paragraph above, this is likely related to the heat capacity of the seeds (see Section 3.2. for discussion). Although the fluid temperature increases relatively quickly, the temperature increase in the seeds lags behind, leading to a temporary condition of relatively cool seeds immersed in a warmer fluid (t = 120 min in Figure 7). Under quasi-stable conditions, the temperature inside the seeds is similar to that of the surrounding fluid, as expected.

For considering the effects of temperature distribution on supersaturation, it is also important to account for the temperature differences between each seed and the nutrient. A driving force for transport away from the seeds can be assumed under the conditions of the etch-back stage even after an initial, approximate saturation of the solution (assuming that solubility is retrograde for the entire temperature range studied). Under the assumption of retrograde solubility, the direction of transport should transition to the opposite direction after about 90 min, as temperatures of seeds and the nutrient become rather similar around this time, and thereafter, the temperatures of seeds increase further, whereas the temperature of the nutrient decreases further.

### 3.1. Quasi-Stable Conditions at Dissolution Stage

At the dissolution stage (left subfigures of Figure 5 and Figure 6) as well as at other times investigated, the temperature distribution is relatively uniform within the nutrient, whereas a noticeable thermal gradient is observable along the vertical direction throughout the growth zone and more generally, in the major free flow regions. This suggests that those thermal gradients are related to convective heat transfer and, consequently, to the flow field. At the top of the autoclave, a smaller flow cell develops, which extends into the upper third of the nutrient and exhibits an upward flow at the center and a downward flow near the vertical autoclave wall. Within the remaining part of the nutrient height, a larger flow cell develops, which exhibits downward flow inside the nutrient and upward flow in the gap between the nutrient and autoclave wall. A circular flow cell is found around the baffle, i.e., the downward flow through the center changes its direction already in the vicinity of the baffle and joins an upward flow passing through the gap between the baffle and autoclave wall. This is likely related to the upward flow near the center that is observed in the growth zone and probably originates from the cold wall in the growth zone at the dissolution stage.

The temperatures of all the seeds, as well as the fluid surrounding them, are in a range of about 440 to 480 °C, which is well above the minimum parameters required for the dissolution of GaN with NH_4_F mineralizer (about 220 °C, 21 MPa) [21]. Therefore, back etching of the seeds can be expected to occur at this stage as anticipated. Due to the higher temperature and thus faster reaction kinetics and earlier onset of etch-back, the amount of etch-back can be expected to be highest for the top seed and lowest for the bottom seed. In the nutrient region, the temperature is significantly higher (slightly below 520 °C). Therefore, the dissolution rates in the nutrient region are likely higher than in the growth zone until a saturated solution is established within the upper zone of the autoclave, which is also fostered by the larger surface area of the nutrient. The inverted flow direction in the lower zone, as well as the overall slow convection speeds during etch-back, are thought to prevent the transport of the saturating and eventually saturated solution to the seeds, hereby ensuring sufficient back etching of the seeds prior to growth. Note, however, that the here-studied convection driven by thermal gradients will be superimposed by solutal convection in an actual growth experiment. Due to the presumably faster dissolution in the upper zone, the density of the solute-containing fluid there will likely increase faster compared to the density of the fluid in the growth zone. This inequal density increase is expected to increase the driving force for downward flow into the growth zone. However, the increasing concentration of Ga-containing solutes formed with NH_4_F mineralizer is also suspected to increase the viscosity [22], which might counteract the mentioned effect of density increase.

The velocity magnitudes at the dissolution stage are below 0.007 m/s, with much lower velocity magnitudes in most regions (see Figure 6 and Figure A1). Only in the uppermost region within and especially above the nutrient, velocity magnitudes comparable to those at the growth stage are found. This is expectable because the nutrient zone has a higher temperature than the top of the autoclave, and therefore, a driving force for natural convection exists in the uppermost part of the autoclave. The temperature of the growth zone, however, is lower than that of the nutrient region above, and therefore there is no local driving force to cause pronounced natural convection within the growth zone at the dissolution stage.

### 3.2. Transition from Quasi-Stable Conditions at Dissolution Stage to Growth Stage

**Figure 8:**
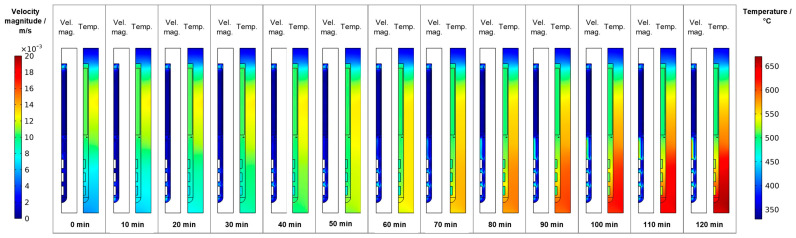
Velocity magnitude and temperature fields occurring during set temperature change for temperature inversion (0 min: quasi-stable etch-back condition, 120 min: reaching of growth stage set temperatures).

Following the gradual change of set temperatures during the temperature inversion process, the downward flow from baffle to first seed gradually develops, mostly during the second half of the temperature inversion process (velocity magnitude subfigures in Figure 8). The strengthened downward flow in the center brings cooler fluid into the growth zone, which counteracts the fluid heating effect of the increasingly warm autoclave walls there. As a result of these effects, increasing temperature differences between the seeds and the surrounding fluid develop, as well as increasing temperature differences between the autoclave wall and the surrounding fluid. Both can be seen by comparing the thermal fields at different times of the temperature inversion process shown in the temperature subfigures in Figure 8. These effects are likely exacerbated by differences in volumetric heat capacity (GaN: 3.19 MJ∙K^−1^∙kg^−3^, Ni-Cr superalloy: 4.4 MJ∙K^−1^∙kg^−3^, supercritical NH_3_: 0.99 MJ∙K^−1^∙kg^−3^, all based on values for specific heat capacity and density as stated in [17]). That is, the lower volumetric heat capacity of ammonia in comparison to GaN leads to relatively quick changes in fluid temperature followed by a sluggish response of the GaN components immersed in the fluid. Note that the fluid in the growth zone exhibits a temperature that is in-between the temperature of seeds and the autoclave wall at the same vertical position. That is, autoclave walls already show higher temperatures than the fluid, but the temperature inside the seeds is temporarily lower than that of the surrounding fluid. Therefore, parasitic nucleation on the autoclave walls is likely to occur, especially during the first 90 min of the set temperature inversion process. Starting from 90 min, the temperatures of the seeds rise above that of the nutrient, which is expected to lead to deposition on the seeds. However, parasitic nucleation or growth on the growth zone inner walls is likely to continue simultaneously as the temperature of the autoclave walls is even higher.

**Figure 9:**
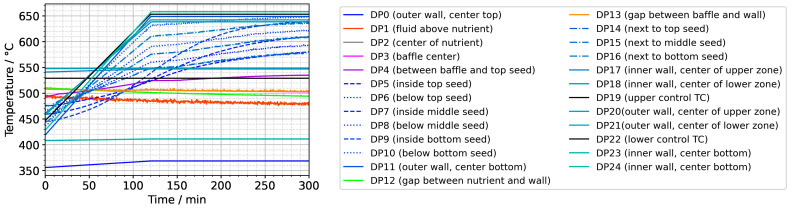
Temperatures over time for the entire set of domain probes.

For all domain probe locations, the resulting temperatures plotted over time are presented in Figure 9. Significant short-term fluctuations are visible for DP1 (at the center halfway between the top of the nutrient and the top inner wall) and DP13 (in the gap between the baffle and autoclave wall). Note that the temperature data for DP3 (in the center of the center hole of the baffle) are hidden by the data of DP13 but exhibit significantly fewer short-term fluctuations, similar to DP4 (between the baffle and top seed in the center). At present, it is not entirely clear whether the oscillations in temperatures in the gap regions represent a physical effect or whether numerical artifacts related to the mesh in this narrow region play a noticeable role. The absence of significant temperature fluctuations for the other domain probes deviates from previously published experimental results of one of the authors [10], which is, however, explainable due to significant differences in the experimental setup and procedures. Firstly, the experimental setup used in that previous study [10] contained a much smaller volume fraction of solids in the nutrient zone, which represented much less of an obstacle to natural convection. Secondly, the control thermocouples in that setup were located inside the furnace insulation and consequently without thermal contact with the outer autoclave wall (contrary to the here-considered setup). In the previous experimental study [10], fluctuations in autoclave wall temperature (that is, thermal boundary conditions) may therefore have occurred due to possible oscillations in heater powers.

In addition, Figure 9 reveals that the fluid in the growth zone follows the changes in thermal boundary conditions rather swiftly. The bulk material of the seeds in contact with the fluid, however, shows a more sluggish, damped response to the imposed boundary condition change (see DP5 to DP10). As elaborated before, this is likely related to the relatively high heat capacity of the crystals slowing down their temperature increase.

**Figure 10:**
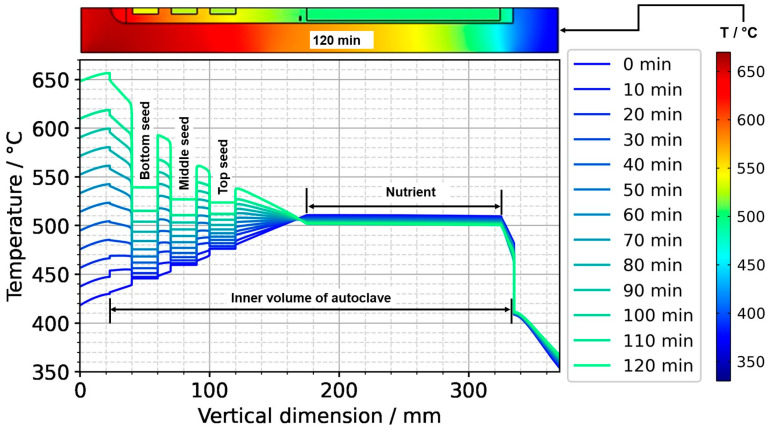
Temperature distribution in vertical direction in the center (0 min: quasi-stable etch-back condition, 120 min: reaching of growth stage set temperatures).

**Figure 11:**
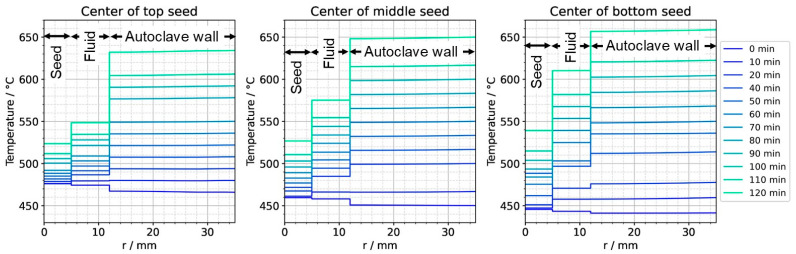
Temperature distribution in horizontal direction through the center of each seed (0 min: quasi-stable etch-back condition, 120 min: reaching of growth stage set temperatures).

To further ease quantitative interpretation at various times of the set temperature inversion process, a vertical temperature profile along the centerline of the autoclave is presented in Figure 10 for the respective times. Horizontal temperature profiles through the center of each seed are presented in Figure 11. At quasi-stable etch-back condition (0 min), the temperature of each seed is an average of the temperatures of the surrounding fluid. Upon reaching growth stage set temperatures (120 min), that is, before re-establishing quasi-stable conditions, the temperatures of the seeds are significantly *lower* than the temperatures of the surrounding fluid: about 70 K for the bottom seed, 45 K for the middle seed, and 20 K for the top seed, respectively. On the contrary, the temperatures of the inner autoclave wall are significantly *higher* than the fluid temperatures: 48 K for the bottom seed, 74 K for the middle seed, and 80 K for the top seed, respectively. That is, the driving force for parasitic growth should be highest at the bottom of the autoclave, as well as the driving force for a possibly ongoing dissolution of the seed (if parasitic deposition temporarily leads to an undersaturated fluid there). The increasing amount of parasitic deposition towards the bottom of the autoclave is in line with typical experimental observations.

Note also that the thermal gradient from one seed to another (slope of the temperature in fluid sections within the growth zone in Figure 10) is also inverted during a set temperature inversion. This occurs after all seeds temporarily reach nearly the same temperature of about 500 °C after about 90 min. The maximum rate of temperature change of seeds is observed for the bottom seed directly before growth stage set temperatures are reached: From the time 110 min to 120 min, rates of temperature change are about 2.5 K/min for the bottom seed, 1.8 K/min for the middle seed, and 1.2 K/min for the top seed, respectively. The fastest rates of seed temperature change correlate with the highest temperature differences between each seed and its fluid surrounding, which is in accordance with expectations because the temperature difference drives these temperature changes.

### 3.3. Re-Establishment of Quasi-Stable Internal Conditions after Set Temperature Inversion

**Figure 12:**
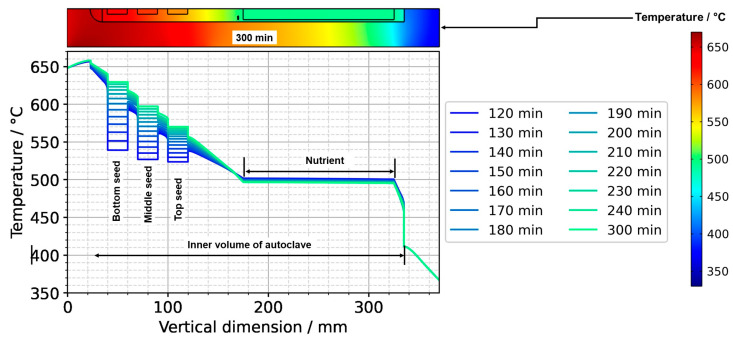
Temperature distribution in vertical direction in the center (120 min: reaching of growth stage set temperatures, 300 min quasi-stable conditions of growth stage).

**Figure 13:**
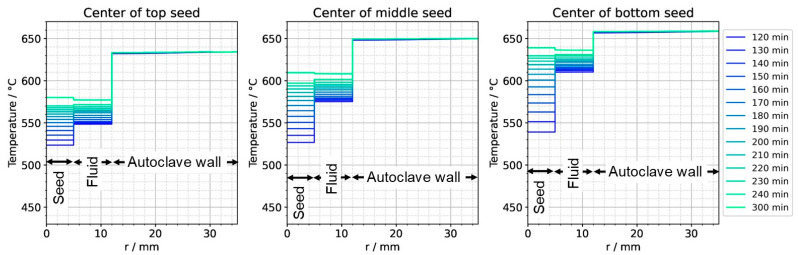
Temperature distribution in horizontal direction through the center of each seed (120 min: reaching of growth stage set temperatures, 300 min quasi-stable conditions of growth stage).

After the process of set temperature inversion is completed, it takes about 3 h for the system to reach quasi-stable conditions again. The respective temporarily occurring temperature distributions, as well as the one under quasi-stable conditions of the growth stage, are shown in Figure 12, alongside the quasi-stable growth stage temperature distribution. Expectably, the rates of temperature change decrease gradually as quasi-stable conditions are approached. After about 2 h, the seed temperatures become an average of the temperatures of the surrounding fluid (Figure 12 and Figure 13).

Figure 13 also reveals the respective temperature differences between the autoclave wall and the interior of the autoclave at the same vertical position. The temperature differences between the autoclave wall and fluid do not decrease nearly as much as the temperature difference between seeds and fluid. However, the significant downward flow of saturated fluid in the center region of the autoclave is thought to favor the deposition on the seeds already at this stage of the process.

**Figure A3:**
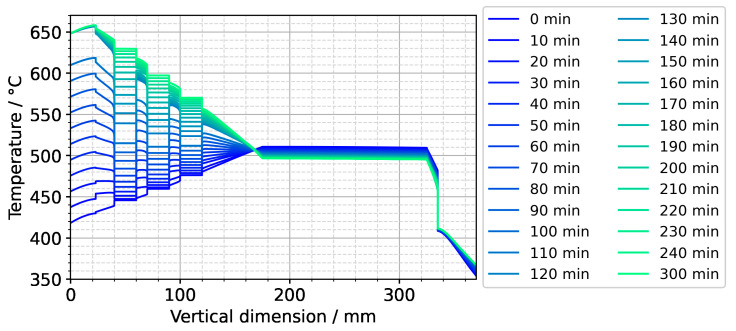
Temperature distribution along the centerline for the entire set temperature inversion process. The quasi-stable conditions of etch-back stage are present at the time 0 min. The time 120 min represents the moment at which set temperatures have reached their values for growth stage. The time 300 min corresponds to quasi-stable conditions of growth stage.

**Figure A4:**
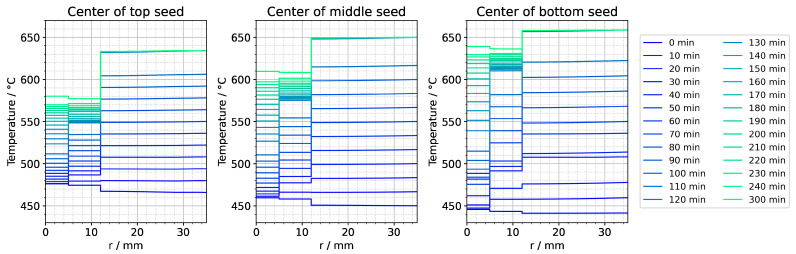
Temperature distribution in horizontal direction through the center of each seed for the entire set temperature inversion process. The quasi-stable conditions of etch-back stage are present at the time 0 min. The time 120 min represents the moment at which set temperatures have reached their values for growth stage. The time 300 min corresponds to quasi-stable conditions of growth stage.

A comprehensive representation containing the temperature distribution data over the entire investigated timespan can be found in Figure A3 (along the centerline) and Figure A4 (horizontally through the seeds).

### 3.4. Quasi-Stable Conditions at Growth Stage

At the growth stage (right subfigures of Figure 5 and Figure 6), a relatively uniform temperature distribution is maintained within the nutrient, and the clockwise flow direction of the upper as well as the counterclockwise flow direction of the main flow cell are maintained. The downward flow extending through the center hole of the baffle is much stronger at the growth stage. In the growth zone, the uppermost flow cell extends from the baffle to the first seed, joining the downward flow from the center hole of the baffle. Along and between the seeds, the downward flow near the center persists. Circular counterclockwise flow cells are observed in between the seeds and the autoclave wall, with the center of the flow cells residing at vertical positions between the seeds, similar to the dissolution stage but with the opposite flow direction.

The thermal gradient along the autoclave wall translates into a thermal gradient inside the autoclave that is modified and shifted by the effects of convection. Consequently, each seed at a different vertical position experiences a different temperature, with temperature differences between seeds of about 15 K. A different temperature of seeds placed at different vertical positions may at least partially explain the experimental observation of growth results depending on the vertical position of seeds. Variations of growth rate depending on the vertical position of seeds have been observed not only in our own experiments using the ammonoacidic mineralizer NH_4_F but were also reported in a detailed analysis of ammonobasic experiments by Grabianska et al. [20]. Under quasi-stable conditions of the growth stage, temperature differences between fluid and seed reach up to about 10 K at the upper and lower ends of the bottom seed (Figure 12), with the seeds being in contact with warmer fluid at their lower end and with cooler fluid at their upper end. While temperature differences between seed and fluid are negligible at the center of the seed, there are significant temperature differences between fluid and autoclave wall. These differ significantly at the vertical positions of each seed (about 60 K next to the top seed, 50 K next to the middle seed, and 30 K next to the bottom seed, with fluid temperatures being lower than the autoclave wall temperatures in all cases, see Figure 13). However, the direction of flow cells supports the deposition on the seeds, as fluid coming from the nutrient zone tends to reach the seeds before coming in contact with the autoclave walls.

Velocity magnitudes at the growth stage reach up to about 0.02 m/s. The highest velocity magnitudes are found around the centerline in-between the baffle and the uppermost seed. At this location, the velocity magnitude at the growth stage is about ten times the velocity magnitude at the dissolution stage. The lowest velocity magnitudes are observed in the nutrient region and partially in the growth zone (see Figure 6).

### 3.5. Lateral Temperature Distribution within the Autoclave Wall

**Figure 14:**
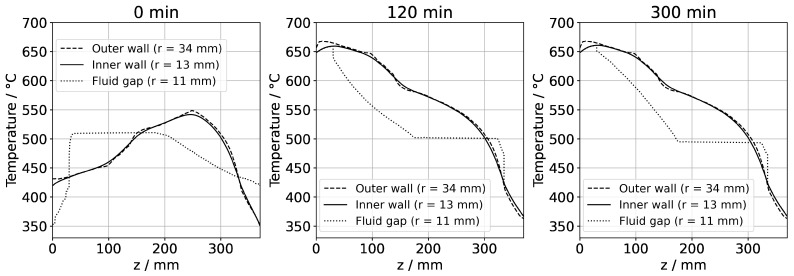
Temperatures inside the autoclave wall near the outer (dashed lines) and inner (solid lines) walls and inside the fluid near the inner autoclave wall (dotted lines). The data labeled with 0 min correspond to quasi-stable conditions at dissolution stage, whereas the data labeled with 120 min correspond to the time at which growth stage set temperatures were reached. The data labeled with 300 min correspond to quasi-stable conditions at growth stage.

In the following, the impact of convective heat transfer on the temperatures of the inner autoclave walls shall be evaluated for quasi-stable conditions as well as for transient conditions with typical rates of set temperature change. For this purpose, temperatures near the inner and outer autoclave wall along the vertical dimension of the simulation domain are displayed in Figure 14. In addition, the respective temperature profile in the fluid near the inner wall is shown for comparison. The radial position of r = 11 mm corresponds to the middle of the gap between the nutrient and the inner wall. Inside the autoclave wall, the differences between temperatures near the outer and inner walls are small, suggesting that meaningful results could likely also be achieved if imposing the outer wall thermal boundary conditions at or much closer to the inner autoclave wall. From our previous results obtained with a simulation of the entire furnace [17], this had not been obvious, as more significant temperature differences between outer and inner walls had been observed there. This was likely an inaccuracy in the previous simulation that originated from a less precise simulation of the convective flow in the sense of not resolving flow phenomena at small time scales (a time resolution comparable to the present study was not feasible in the previous study due to the large domain size and need to perform the calculation for a high number of heater power combinations). In agreement with this interpretation of the deviation, the flow cells observed in the previous study are also somewhat different from those found in the present study. The time resolution was improved in the present simulation using much smaller timesteps. We conclude that while it is necessary to use continuous temperature distributions reflecting realistic thermal losses, it is likely not necessary to include the full thickness of the autoclave wall in the simulation domain if set temperature changes occur gradually.

### 3.6. Experimental Results Obtained Using Conditions Similar to the Numerical Simulation

**Figure A5:**
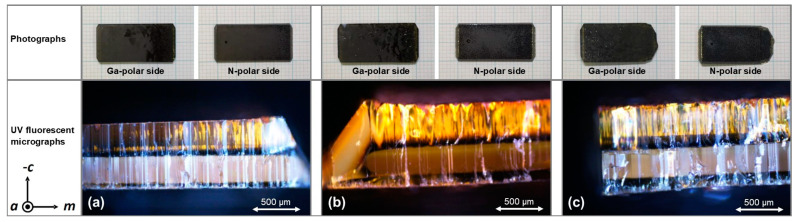
Photographs (top) and UV fluorescent micrographs of cleavage planes (bottom) of crystals grown at (**a**) top, (**b**) middle, and (**c**) bottom position. The lack of optical transparency of the crystals visible in the photographs is related to the cost-effective choice of seeds (HVPE) and nutrient due to the different goals of this study. The UV micrographs were used to quantify seed thickness loss by etch-back as well as effective growth rates stated in Table 8.

The UV fluorescent micrographs of the cleaved crystals are presented in Figure A5. The interfaces of seeds and grown layers are clearly visible as intended. The experimental results in terms of GaN mass transport are listed in Table 7 and Table 8 in terms of mass changes, dimensional changes, and related quantities, respectively. As expected, the overall mass changes indicate transport from the nutrient to the growth zone. The top seed shows the smallest increase in mass; however, this seed has also experienced the largest loss in thickness by etch-back and hence the largest (estimated) mass loss by etch-back. The effects of seed positioning will therefore be discussed based on the remaining seed thicknesses and the effective growth rates (that is, considering growth from the seed interface rather than the thickness change during the overall experiment).

The amount of seed thickness loss increases from the bottom to the top seed. This may be due to the different seed temperatures during etch-back, as the seed temperatures increase from the bottom to the top seed. The different temperatures are thought to influence dissolution kinetics: dissolution is generally expected to be faster at higher temperatures. This should lead to a larger amount of thickness loss at the upper seeds within a given timespan. Assuming a retrograde solubility also in the temperature range used for etch-back, mass transport should occur from bottom to top seed, which could slow down the etch-back of the upper seeds and facilitate the etch-back of the lower seeds. This may contribute to the experimentally observed dissolution of the bottom part of the bottom seed. Since seed mass loss is highest for the uppermost seed, etch-back appears to be dominated by dissolution kinetics rather than by the temperature dependence of solubility, which is expectable because, initially, there is no saturated solution.

The effective cumulative growth rates increase towards the bottom seed position. This trend has also been seen in other experiments with similar configurations. The increase in effective cumulative growth rates coincides with the increase in seed temperatures from the top to the bottom position at the growth stage. This is likely due to the increasing temperature difference between the nutrient and the respective seed. Effective face-specific growth rates fit this trend on both the Ga- and N-face. On all seeds, growth on the N-face was faster than on the Ga-face. However, the ratio of face-specific growth rates decreased towards the bottom of the autoclave.

On the N-face of each crystal, two sublayers can be distinguished within the grown layer (see UV fluorescent micrographs in Figure A5). The ratio of sublayer thicknesses changes with the vertical position of the seed in the autoclave as follows. For the uppermost seed, a ratio of complete N-face layer thickness to dark N-face sublayer thickness of about 6.0 is observed. This ratio decreases to about 4.8 for the middle and about 3.4 for the bottom seed. The absolute thickness of the dark sublayer also increases towards the bottom of the autoclave (top seed: 61 µm, middle seed: 93 µm, bottom seed: 140 µm). Since the distinguishable sublayers must be expected to represent distinguishable growth conditions occurring within distinguishable timeframes during the experiment, we suspect that the dark sublayer may represent the material grown while seed temperatures were still significantly lower than at quasi-stable conditions of the growth stage. Since the duration of etch-back was likely sufficient to establish a saturated solution also within the growth zone (as discussed in Section 2.2), we expect growth to be initiated by the rise of fluid temperature, which begins early during the set temperature inversion process (according to the numerical simulation, see Figure 10). The timespan with significant differences between the temperature of each seed and its fluid surrounding can be estimated to be about 4 h according to the simulation (2 h for the actual set temperature inversion process and another 2 h with diminishing thermal gradients between crystals and surrounding fluid), whereas the quasi-stable conditions of growth stage are established after about 5 h in total.

All in all, the experimental and numerical results can be interpreted in a coherent way, supporting the correctness of the main characteristics determined via numerical simulations.

## 4. Conclusions

As expected, both numerical and experimental results indicate a systematic variation of growth conditions along the vertical axis of the autoclave. According to the analysis of cleavage surfaces of the grown crystals under UV illumination, the effective cumulative growth rates increase towards the bottom of the autoclave. According to the numerical model, this correlates with an increasing temperature at the growth stage. Moreover, according to the analysis of the cleavage surfaces, the amount of etch-back (seed thickness loss) decreases towards the bottom of the autoclave. According to the numerical model, this correlates with a decreasing temperature at the dissolution stage towards the bottom of the autoclave. Therefore, the transport and recrystallization process of crystal growth appears to be governed mostly by a retrograde solubility, whereas the process of etch-back appears to be strongly influenced by reaction kinetics, provided that the solubility of GaN is retrograde under both the dissolution stage and growth stage conditions investigated.

According to the numerical results, short-term temperature fluctuations originate primarily from fluctuations in velocity magnitude, while the flow direction usually exhibits only minor changes within short time scales. However, it should be noted that flow directions might be stabilized by the symmetrical nature of the axis of this 2D model.

Methodically, it can be concluded that if all set temperature changes (more generally: boundary condition changes) are applied gradually and if the initial values are close enough to the solution for the initial boundary condition, it should be feasible to exclude most of the autoclave wall thickness from the simulation domain to reduce computation times.

The numerical model is found to be particularly insightful in terms of analyzing temporary effects, including temperature differences between crystals and the surrounding fluid, which would be extremely difficult to observe experimentally.

The response of the fluid to changes in the autoclave wall temperatures is relatively swift, whereas the response of the crystals is relatively sluggish. This is likely related to the low volumetric heat capacity of the fluid compared to both GaN and the nickel base alloy. The rates of seed temperature change range from 2.5 K/min to 1.2 K/min and are thus closely related to the rate of set temperature change (100 K/h, i.e., about 1.7 K/min). Due to the relatively swift response of the fluid temperature compared to the seeds, the following occurs during the set temperature inversion process: Depending on the vertical position of the seed, significant temperature differences of 20 K to 70 K occur temporarily between a seed and its fluid surrounding during the set temperature inversion process, so that the fluid temperature in the growth zone is between that of the seeds and that of the neighboring inner autoclave walls. Therefore, in a retrograde solubility scenario, parasitic nucleation and growth on the autoclave walls may already occur while the seeds are not growing yet or may still experience back-etching. This may explain why the bottom part of the lowest seed tends to dissolve preferentially: If the seed is temporarily colder than the fluid and the inner autoclave wall is warmer, then mass transport from the bottom of the lowest seed to the bottom inner wall is expectable. In addition, the relatively high surface area in the vicinity of the lowest seed may exacerbate this effect, as parasitic deposition at the bottom autoclave wall may lead to an undersaturated solution in its vicinity. These insights also pose the question of whether ammonothermal growth with normal and retrograde solubility differ fundamentally in the conditions during the early stages of growth. If a normal solubility configuration is used, the positions of seeds and nutrients are inverted. In this case, it would be the nutrient rather than the seeds that would approach growth condition temperatures in a sluggish way, as the seeds would be residing in the zone that does not experience major changes in thermal boundary conditions. This suggests that if a similar temperature ramp-up process is used for growth under normal solubility conditions, then nucleation on the seeds may occur earlier in the set temperature inversion process. In addition, crystal growth could occur already while the crystals approach their quasi-stable growth condition temperatures, and significant fluid-crystal temperature differences could result in fast growth during the early stages of the process. This might affect structural quality as well as impurity incorporation. Further research is needed to clarify if this is the case and whether there is a general advantage of retrograde solubility process variants due to such effects.

About 90 min into the process of set temperature inversion, the temperatures of the seeds begin to exceed those of the nutrient. Therefore, crystal growth is likely initiated around that time due to the inversion of the solubility gradient. When the process of set temperature inversion is completed after 120 min, the most pronounced thermal gradients between seeds, fluid, and autoclave wall are observed. Within another 120 min (120 min after reaching the growth stage set temperatures), the mean temperature of each seed approaches the average temperature of its fluid surrounding. It takes an additional 60 min until approximately quasi-stable conditions of the growth stage are established.

## Figures and Tables

**Table 1:** Geometry of the model: Components, radial, and vertical positions referring to the center of each component, and radial width as well as vertical height of the components. For the seeds, the thickness refers to the model version with the thicker seeds.

Component	Position r/mm	Δr/mm	Position z/mm	Δz/mm
Autoclave	0.0	35.0	185.0	370.0
Nutrient	0.0	10.0	250.0	150.0
Baffle	8.3	3.4	170.0	1.0
Top seed	0.0	5.0	110.0	20.0
Middle seed	0.0	5.0	80.0	20.0
Bottom seed	0.0	5.0	50.0	20.0
Inner volume of autoclave	0.0	12.0	185.0	300.0

**Table 2:** Detailed description of domain probes, including the labels used, radial and vertical position, and description of locations.

Label	Position		Description of Location: Radial Position, Vertical Position (Further Description)
	r/mm	z/mm	
DP0	0.00	369.00	Center, 34 mm from top inner wall (near upper outer autoclave wall)
DP1	0.00	330.00	Center, above nutrient halfway between inner upper autoclave wall and top of nutrient
DP2	0.00	250.00	Center of the nutrient
DP3	0.00	170.00	Center, at half baffle height
DP4	0.00	145.00	Center, halfway between bottom edge of the baffle and upper edge of the top seed
DP5	0.00	110.00	Center of the top seed
DP6	0.00	95.00	Center, in the middle between top and middle seed
DP7	0.00	80.00	Center of the middle seed
DP8	0.00	65.00	Center, in the middle between middle and bottom seed
DP9	0.00	50.00	Center of the bottom seed
DP10	0.00	35.00	Center, between bottom seed and bottom inner wall
DP11	0.00	1.00	Center, 34 mm from bottom inner wall (near outer autoclave wall)
DP12	11.00	250.00	Middle of gap between nutrient and inner wall, half nutrient height
DP13	11.00	170.00	Middle of gap between baffle and inner autoclave wall, half baffle height
DP14	6.75	110.00	Halfway between edge of top seed and inner autoclave wall, center of top seed
DP15	6.75	80.00	Halfway between edge of middle seed and inner autoclave wall, center of middle seed
DP16	6.75	50.00	Halfway between edge of bottom seed and inner autoclave wall, center of bottom seed
DP17	13.00	252.50	1 mm from inner autoclave wall, center of upper zone (near inner autoclave wall)
DP18	13.00	102.50	1 mm from inner autoclave wall, center of lower zone (near inner autoclave wall)
DP19	34.00	280.00	1 mm from outer autoclave wall, control thermocouple of upper zone
DP20	34.00	252.50	1 mm from outer autoclave wall, center of upper zone
DP21	34.00	102.50	1 mm from outer autoclave wall, center of lower zone
DP22	34.00	70.00	1 mm from outer autoclave wall, control thermocouple of lower zone
DP23	0.00	22.00	Center, 1 mm from inner autoclave wall (near bottom inner wall)
DP24	0.00	336.00	Center, 1 mm from inner autoclave wall (near top inner wall)

**Table 3:** Components with the numerical label used in Figure 1 and description of the respective materials.

Component (Number)	Description
NH_3_ (1)	At 426.6 °C, 100 MPa, treated as incompressible
Autoclave wall (2)	Ni-Cr superalloy
Baffle (3)	Corrosion-resistant metal
Bulk GaN (4)	Wurtzite GaN

**Table 6:** Parameters applied for mesh generation.

	Maximum Element Size/mm	Minimum Element Size/mm	Maximum Element Growth Rate	Curvature Factor	Resolution of Narrow Regions
Autoclave walls	122.00	18.5000	2.00	1.0	0.9
Free flow	1.00	0.1000	1.15	0.6	0.8
Baffle	0.25	0.0074	1.10	0.2	1.0
Nutrient	1.23	0.0350	1.13	0.3	1.0
Seeds	0.80	0.0074	1.13	0.2	1.0

**Table 7:** Masses of GaN components before and after the ammonothermal growth experiment. Changes with negative values represent a decrease in mass. Note that the bottom edges of the bottom seed (position 15 mm) dissolved completely so that the surface area available for growth on the bottom seed was reduced.

GaN Component	Pre-Run Mass/g	Etch-Back Mass Loss (Estimate)/g	Post-Run Mass/g	Mass Change/g	Mass Change/%
Nutrient	35.59	Not determined	14.14	−21.45	−60.3
Top seed (58 mm)	0.4394	−0.1098	0.9517	0.5123	116.6
Middle seed (35 mm)	0.4397	−0.0892	1.1323	0.6926	157.5
Bottom seed (15 mm)	0.4399	−0.0795	1.0679	0.6280	142.8

**Table 8:** Results of the growth experiment. Note that face-specific growth rates refer to the polarity of the seed, which could potentially differ from the polarity of the grown layer in case of polarity inversion. Note as well that the bottom edges of the bottom seed (position 15 mm) dissolved. Note that growth rates were determined based on the duration of constant growth stage set temperatures, which is only an approximation of the actual timespan during which growth occurs.

Seed Position/mm	Seed Thickness Loss/µm	Effective Ga-Face Growth Rate/µm/d	Effective N-Face Growth Rate/µm/d	N:Ga Face Growth Rate Ratio	Effective Cumulative Growth Rate/µm/d
58	99	14.3	86.5	6.1	130.3
35	83	30.5	107.8	3.5	168.8
15	74	36.5	113.8	3.1	176.5

## Data Availability

The data presented in this study are available upon reasonable request from the corresponding author.

## Supplementary Materials

The following supporting information can be downloaded at: https://zenodo.org/record/7555506#.Y8rq5HbMKUk, Video S1: dissolution stage (upper part of nutrient and above), Video S2: dissolution stage (baffle region), Video S3: dissolution stage (top seed), Video S4: dissolution stage (middle seed), Video S5: dissolution stage (bottom seed), Video S6: growth stage (upper part of nutrient and above), Video S7: growth stage (baffle region), Video S8: growth stage (top seed), Video S9: growth stage (middle seed), Video S10: growth stage (bottom seed).
